# Factors Influencing the Timeliness and Completeness of Appropriate Staging Investigations for Patients with Stage I–III Lung Cancer in Southeastern Ontario

**DOI:** 10.3390/curroncol31100453

**Published:** 2024-10-11

**Authors:** Shahad AlGhamdi, Nilah Ahimsadasan, Weidong Kong, Michael Brundage, Elizabeth A. Eisenhauer, Christopher M. Parker, Andrew Robinson, Andrew Giles, Geneviève C. Digby

**Affiliations:** 1 Department of Medicine, Division of Respirology, Queen’s University, Kingston, ON K7L 2V7, Canada; 2 School of Medicine, Queen’s University, Kingston, ON K7L 2V7, Canada; 3 Cancer Care and Epidemiology Research Unit, Queen’s University, Kingston, ON K7L 2V7, Canada; 4 Department of Oncology, Queen’s University, Kingston, ON K7L 2V7, Canada; 5 Department of Critical Care Medicine, Queen’s University, Kingston, ON K7L 2V7, Canada; 6 Department of Surgery, Division of Thoracic surgery, Queen’s University, Kingston, ON K7L 2V7, Canada

**Keywords:** lung cancer, multidisciplinary clinic, staging, system barriers

## Abstract

(1) Background: Comprehensive and timely lung cancer (LC) staging is essential for prognosis and management. The Lung Diagnostic Assessment Program (LDAP) in Southeastern (SE) Ontario aims to provide rapid, guideline-concordant care for suspected LC patients. We evaluated factors affecting the completeness and timeliness of staging for stage I–III LC patients in SE Ontario, including the impact of LDAP management. (2) Methods: This was a population-based retrospective cohort study using the LDAP database (January 2017–December 2019), linked with the Ontario Cancer Registry, to identify newly diagnosed LC patients. A Cox model approach identified variables associated with staging completeness and timeliness. (3) Results: Among 755 patients, 459 (60.8%) were managed through LDAP. Optimal staging was achieved in 596 patients (78.9%), 23 (3.0%) had alternative staging, and 136 (18.0%) had incomplete staging. In the adjusted analyses, LDAP management was associated with a higher likelihood of complete staging (OR 2.29, *p* < 0.0001) and faster staging completion (β = −18.53, *p* < 0.0001). Increased distance to PET centres was associated with a longer time to complete staging (β = 8.95 per 100 km, *p* = 0.0007), as was longer time to diagnosis (β = 21.63 per 30 days, *p* < 0.0001). (4) Conclusions: LDAP management in SE Ontario significantly improved staging completeness and shortened staging time for stage I–III LC patients.

## 1. Introduction

Lung cancer (LC) represents a significant health system challenge as the leading cause of cancer-related mortality worldwide [1]. It is an aggressive malignancy with a high mortality rate and most cases are diagnosed at an advanced stage [1,2]. Delays in diagnosis and staging can further worsen prognosis by hindering treatment initiation, highlighting the importance of expedited and comprehensive diagnostic and staging processes to optimize survival outcomes [3,4].

Within Canada, marked disparities in LC outcomes are evident, with pronounced differences observed across geographic regions and demographic groups [5]. Even within the province of Ontario, there is significant variability in LC outcomes, with Southeastern Ontario (SE) having a lower 5-year LC survival [5,6]. These discrepancies are partly attributed to socioeconomic and geographic factors, with vulnerable populations (including those of lower socioeconomic status, rural residents, recent immigrants, and Indigenous communities) bearing a disproportionate burden of the disease [7].

A crucial aspect of LC management is comprehensive and timely staging. Prolonging the time to complete diagnostic work-up or treatment planning can lead to disease progression, detrimentally impacting prognosis [8,9]. Lung Diagnostic Assessment Programs (LDAPs) are rapid multidisciplinary assessment programmes that provide efficient and appropriate diagnostic and staging work-up for patients with suspected LC [10,11,12]. LDAP-based management has been associated with improved time to diagnosis, specialist assessment, and treatment [11,13], and often includes patient navigation, psychosocial support, and a multidisciplinary team approach involving different specialists [14,15,16]. In Southeastern Ontario, LDAP management in the diagnostic phase is associated with improved 2-year LC survival compared to patients not managed by the LDAP [13]. Increased geographical distance from the LDAP is associated with a lower probability of LDAP management [13].

Recognizing the importance of timely and comprehensive LC staging on outcomes, the SE LDAP implemented a quality improvement initiative in 2018 to expedite staging investigations through standardized triage pathways, which led to faster times to PET scans and brain imaging [10]. Despite this, time to staging completion remains lengthy, and patients from our region must travel long distances to access some staging investigations. To further understand the factors that contribute to the variability in outcomes within SE Ontario, we sought to elucidate the factors influencing completeness and timeliness of staging investigations for patients with stage I–III LC to identify opportunities for improvement that specifically target identified barriers to care.

## 2. Materials and Methods

### 2.1. Local Context

Southeastern Ontario (SE Ontario) is a largely rural region situated to the east of Toronto and southwest of Ottawa along the northeastern shore of Lake Ontario in Canada. Its largest city, Kingston, had a population of ~135,000 in 2017, at which time the population of the entire region was estimated at 500,000 people, or 3.6% of the Ontario population [17]. Approximately 60% of the patients with LC in SE Ontario undergo evaluation through the SE LDAP. This LDAP is managed by respirology and thoracic surgery specialists, with care coordination by nurse navigators, and streamlines the diagnostic and staging processes from the time of referral to the initial oncological assessment and treatment. LDAP nurse navigators connect with patients at the time of referral, coordinate staging investigations requested by physicians at referral triage, and work with patients to overcome transportation challenges to attend testing, as required.

Due to the rural geography of SE Ontario, diagnostic and imaging facilities are sparsely located. Until recently, MRI was only available in 2 cities within the health region, 82 km apart. There is no PET-CT scanner in SE Ontario; patients must travel to adjacent health regions in Ottawa and Toronto, with the closest PET-CT located 200 km from the SE regional LDAP. Invasive mediastinal staging by EBUS or mediastinoscopy is only available at the regional tertiary care centre at Kingston Health Sciences Centre.

### 2.2. Study Design and Databases

We conducted a population-based retrospective cohort study from 1 January 2017 to 31 December 2019. We identified patients with newly diagnosed LC through the Ontario Cancer Registry (OCR). We then linked these data to the SE LDAP database to identify patients who received care through the LDAP.

Descriptive data were collected, including patient characteristics (age, sex, and income quintile) and disease characteristics (histologic subtype and stage). Staging investigations included imaging studies completed (PET–CT, brain imaging, nuclear medicine bone scan, and CT abdomen +/− pelvis), invasive mediastinal staging procedures (EBUS and mediastinoscopy), and first diagnostic procedure (CT-guided biopsy, EBUS bronchoscopy, and others).

Timeliness of care metrics included time to diagnosis (defined as time from first abnormal CT chest imaging to diagnosis), and time to completion of PET-CT and staging investigations (defined from time of first abnormal CT chest imaging). We also evaluated the influence of LDAP utilization on the completeness and timeliness of staging investigation compared to those diagnosed outside of the LDAP. Data are presented descriptively as means and N (percent).

### 2.3. Case Definitions, Demographics, and Study Outcomes

This study included individuals aged 18 years and older who received a new diagnosis of LC between 1 January 2017, and 31 December 2019. Diagnosis was determined by initial topography and morphology coding consistent with LC in OCR. In the analysis, lung cancer histology was categorized into adenocarcinoma and non-adenocarcinoma subtypes (including squamous cell, poorly differentiated carcinoma, small cell carcinoma, large cell carcinoma, neuroendocrine carcinoma not otherwise specified, and other).

Exclusion criteria included patients lacking data in OCR, LC recurrence (i.e., not a new diagnosis), diagnosis outside the specified study period, non-LC histology type, or individuals residing outside the SE Ontario region. We also excluded patients with stage IV or unknown LC stage due to the lack of data regarding stage migration and inability to differentiate patients who had stage IV disease at the time of first clinical assessment from those who were found to have stage IV disease only after completing staging.

Complete staging was defined as the number (%) of patients that completed appropriate (optimal or alternative) staging during the diagnostic phase based on the American College of Chest Physicians 2013 guidelines [18], as shown in Table 1.

We report the number and percentage of patients that completed appropriate staging (either optimal or alternative), as well as the number and percentage of patients who completed optimal staging and those who completed alternative staging.

### 2.4. Statistical Approach

In our analysis, odds ratios (ORs) were used to describe the effect of factors in model-based analyses completeness of staging investigation, and beta coefficients (β) were reported to describe the effect of factors on time to complete staging investigation along with their corresponding *p*-values. A *p*-value < 0.05 was deemed as statistically significant.

## 3. Results

A total of 755 patients with newly diagnosed stage I–III LC were identified from the OCR database. Of these, 459 patients (60.8%) were managed through LDAP and 296 patients (39.2%) were managed by non-LDAP processes.

### 3.1. Patient and Disease Characteristics

Patient and disease characteristics at the time of LC diagnosis are summarized in Table 2.

Among the 755 identified patients with stage I–III LC, the median age was 72 years and 58% were female. The majority of patients were in the second and third income quintiles (220 [29.1%] and 250 [33.1%], respectively) (Table 2). At the time of diagnosis, 382 patients (50.6%) had stage I LC, 106 (14.0%) had stage II disease, and 267 (35.4%) had stage III disease. In terms of LC histology, 329 (43.6%) had adenocarcinoma (Table 2).

### 3.2. Staging Investigations for Patients with Stage I–III Lung Cancer

#### 3.2.1. Completeness of Staging Investigations

During the diagnostic phase, a total of 596 patients (78.9%) with stage I–III completed optimal staging investigations, 23 (3.0%) completed alternative staging investigations, and 136 (18.0%) had incomplete staging. Optimal staging investigations were completed in 327 patients (85.6%) with stage I disease and 269 patients (72.1%) with stage II/III disease (*p* < 0.0001). Incomplete staging was observed in 43 patients (11.3%) with stage I disease and 93 patients (24.9%) with stage II/III disease, while 12 patients (3.1%) with stage I disease and 11 patients (2.9%) with stage II/III completed alternative staging (*p* < 0.0001).

Of patients managed through the LDAP, 391 (85.2%) completed optimal staging, compared with 205 (69.3%) of non-LDAP-managed patients (*p* < 0.0001) (Table 2).

A total of 273 patients (36.2%) underwent invasive mediastinal staging by either Endobronchial Ultrasound Transbronchial Needle Aspiration (EBUS-TBNA) or mediastinoscopy. Among these, 229 (83.9%) completed optimal staging, while 11 (4.0%) had incomplete staging (*p* = 0.004).

In terms of brain imaging, 537 patients (71.1%) completed brain imaging, of which 450 (83.8%) completed optimal staging and 19 (3.5%) had incomplete staging (*p* < 0.0001) (Table 3).

A total of 607 patients (80.4%) underwent PET-CT imaging. Of the 382 patients with stage I LC, 327 patients (85.6%) had optimal staging by completing PET-CT. Of the 373 patients with stage II/III LC, 270 (75.1%) completed PET-CT.

Patients travelled a median distance of 191 km to reach their chosen PET-CT facility. However, the median distance to their nearest PET centre was 179.3 km. In our cohort, 444 (58.8%) travelled to The Ottawa Hospital (TOH), 154 (20.4%) travelled to other non-TOH PET locations, and 9 (1.4%) had PET in unknown locations (Table 3).

#### 3.2.2. Timeliness of Staging Investigations

For all patients in the cohort, the median time from the first abnormal CT scan to diagnosis was 37 days and the median time from the first abnormal CT scan to the completion of staging investigations was 41 days. The median time from the first abnormal CT scan to PET-CT imaging was 36 days (Table 4).

For patients with stage I LC, the median times to undergo PET-CT imaging and complete staging investigations were both 39 days. For patients with stage II/III LC, the median time to undergo PET-CT imaging was 34 days, while time to complete staging investigations was 42 days (Table 4).

The mean time to complete staging for patients managed outside the LDAP pathway was 53.3 days, compared to 46.6 days for those managed within the LDAP.

### 3.3. Factors Associated with Completeness and Timeliness of Staging Investigation for Patients with Stage I–III Lung Cancer

#### 3.3.1. Completeness of Staging Investigations

Factors associated with the completeness of staging investigations for patients with stage I–III LC are presented in Table 5.

In the adjusted multivariate regression models, age was associated with the likelihood of complete staging (OR 0.79 for age 81+ and OR 1.66 for age 61–70 vs. reference age 18–60, *p* = 0.048). Patient management through LDAP was associated with a higher likelihood of complete staging (OR 2.29 vs. non-LDAP, *p* < 0.0001). Gender, income quintile, and patient distance from the nearest PET centre were not associated with the completeness of staging investigations.

#### 3.3.2. Timeliness of Staging Investigations

Factors associated with the timeliness of staging investigation for patients with stage I–III LC are presented in Table 6.

In the unadjusted analysis, factors associated with shorter time from initial CT scan to complete staging included LDAP management (β = −6.63 versus non-LDAP management, *p* = 0.023) and non-adenocarcinoma histology (β = −11.16 vs. adenocarcinoma, *p* < 0.0001), and the use of bronchoscopy/EBUS as a diagnostic procedure (β = −7.73 vs. CT-guided lung biopsy, *p* = 0.021). Factors associated with a longer time from CT scan to complete staging included a longer time to diagnosis (β = 18.7 per 30 days, *p* < 0.0001).

In the adjusted multivariate analysis, all these factors remained significant, and an additional factor associated with a shorter time from the initial CT scan to complete staging included stage I disease (β = −13.69 vs. stage II/III, *p* < 0.0001). The influence of LDAP patient management became more pronounced in the adjusted analysis (β = −18.53 vs. non-LDAP management, *p* < 0.0001). An additional factor associated with a longer time to complete staging in the multivariate analysis included increased distance to a PET centre (β = 8.95 per 100 km, *p*= 0.0007).

## 4. Discussion

In this study, we evaluated the factors that influence the completeness and timeliness of staging investigations for patients with stage I–III LC in Southeastern Ontario. We found that patient management through the SE Ontario LDAP was associated with a significantly higher likelihood of completing staging investigations and was strongly associated with faster time to complete staging compared with management outside the LDAP. Meanwhile, increased patient distance from a PET-CT facility was associated with a longer time to complete staging, independent of whether patients were managed by the LDAP. Overall, this suggests that the structured and coordinated care provided by the LDAP enhances the efficiency of the staging process, but that barriers to care such as geographic distance to PET-CT are only able to be partially alleviated by the structured care model of the LDAP.

It has been well demonstrated that significant variability in LC outcomes exists [1], even within Canada [5], and across the province of Ontario [6]. This variability is multifactorial, encompassing patient, disease, and system factors that collectively contribute to these differences in outcomes [6]. Despite advancements in treatment options, the overall five-year survival rate for LC remains relatively low [1], emphasizing the importance of prevention, screening [6], and optimizing access and use of rapid assessment pathways for diagnosis and management [10,11]. Rapid assessment clinics have demonstrated efficacy in expediting the diagnostic process, providing guideline-concordant care [12], improving survival rates [11,13], and reducing patient distress during the diagnostic phase [19]. Our study contributes additional evidence to the growing body of literature supporting the benefits of rapid assessment pathways. The findings underscore the importance of rapid assessment clinics in streamlining the diagnostic process, ultimately leading to improved patient outcomes and enhanced healthcare efficiency.

PET-CT is an essential staging investigation for patients with stage I–III LC [20]. This imaging modality is highly sensitive in detecting the spread of malignancy, thereby significantly improving the accuracy of staging [21]. PET-CT not only enhances the precision of staging but also has a high predictive value, which helps avoid unnecessary investigations and procedures [22]. The improved staging accuracy afforded by PET-CT can lead to more appropriate and targeted treatment plans, ultimately enhancing patient outcomes and optimizing healthcare resources [22]. Despite the numerous benefits of PET-CT in accurately staging LC, its availability remains limited, particularly in rural areas [23]. Patients residing in geographically remote locations often face significant challenges, including travelling long distances to access this diagnostic modality. This accessibility barrier disproportionately affects patients from lower socioeconomic backgrounds, who constitute a majority of our study population and often lack the financial means and logistical support necessary for long-distance travel, and exacerbates inequalities in LC outcomes [7].

Interestingly, distance to the nearest PET-CT facility did not emerge as a significant factor affecting the completeness of staging. Meanwhile, increased distance to a PET-CT facility was associated with a longer time to complete staging in the adjusted model, underscoring the critical role that geographic accessibility plays in the timeliness of cancer staging. The increased travel distance likely introduces delays due to logistical challenges, such as transportation availability, travel expenses, and coordination of appointments. These findings suggest that LDAP management may mitigate certain barriers associated with PET distance. This observation could be attributed to the integral role of nurse navigators within LDAPs, ensuring timely access to necessary investigations, and supporting patients in navigating logistical challenges, thereby potentially contributing to completeness of staging procedures [24,25]. These models of care underscore the potential of LDAPs not only in improving clinical outcomes but also in addressing disparities related to healthcare access and resource utilization, particularly in the context of geographic barriers such as PET facility distance. Future research should explore the extent to which comparable care models can address similar barriers to timely staging in other health systems.

We also found that certain patient, disease, and system factors were associated with the timeliness and completeness of staging investigations. Patients with stage I disease generally had a shorter time to complete staging, likely since fewer staging investigations are generally needed for early-stage LC compared with stage II/III disease. Non-adenocarcinoma histology was associated with a shorter time to complete staging, likely due to the fact that patients with more aggressive disease receive expedited care, with a paroxysmal association of faster care with worse survival outcomes [11,13]. System factors such as the type of biopsy also played a role in the timeliness of staging, likely because EBUS-TBNA can serve as both a diagnostic and staging modality, suggesting that there may be value in ensuring appropriate access to this diagnostic test given its potential to expedite timeliness of staging. Many of these factors are non-modifiable; however, the fact that patient management via LDAP had the greatest positive influence on both completeness of staging investigations and timeliness of staging highlights the critical role of this program in optimizing the care of patients with LC. Additionally, the fact that increased time to diagnosis and increased distance to PET scan had the strongest impact on time to completeness of staging, regardless of LDAP involvement, emphasizes the need for prompt and efficient diagnostic workflows to ensure timely completion of staging.

Not only did LDAP-managed patients undergo faster staging by an average of 1 week, but they were also more likely to complete staging investigations. While a 1-week delay in completing staging may seem minor, it can be critical in the context of an aggressive cancer such as LC where delays in care are associated with worse outcomes [26,27]. Together with the observed increase in the completeness of staging, these results may in part explain the finding from our prior study of this cohort that LDAP management is associated with a lower probability of dying at 2 years [13].

### Limitations

One of the limitations of our study is the exclusion of patients with stage IV disease. This was necessary because the Ontario Cancer Registry (OCR) does not provide data that distinguish between patients who had stage IV disease at the time of their first clinical assessment (e.g., metastatic disease at initial presentation) from those who were found to have stage IV disease only after completing staging investigations (e.g., initially suspected early-stage disease that revealed metastases during work-up). Due to this lack of differentiation, we focused our evaluation on patients with stage I–III LC to ensure a more accurate assessment.

Additionally, our study included all brain imaging, irrespective of contrast usage. This was due to the OCR database’s limitations in identifying the presence of contraindications to contrast dye use. This limitation means that our analysis does not account for the potential impact of lack of contrast agent usage on imaging outcomes.

Our study uses data from 2017 to 2019, as the database was initially compiled in 2022 to analyze factors affecting regional variability in survival outcomes. While a limitation is the absence of data beyond 2019, there have been no changes in regional access to staging investigations, including no new PET centres.

Finally, it is recognized that certain patients with stage I disease might necessitate invasive mediastinal staging due to specific clinical indications, such as centrally located tumours, stage IB disease, synchronous tumours, or small cell LC. Unfortunately, the OCR database lacked the granularity required to identify these particular cases. As a result, our study does not differentiate among patients based on the necessity for invasive mediastinal staging.

These limitations highlight the challenges faced in using registry data for comprehensive staging assessments and underscore the need for more detailed clinical data to improve the accuracy and completeness of LC staging studies.

## 5. Conclusions

For patients with stage I–III LC in SE Ontario, LDAP management increased the likelihood of completing staging investigations and shortened staging time, while increased distance to PET-CT was associated with a longer time to complete staging, regardless of LDAP management. Given that lung cancer is an aggressive disease and that delays in staging have been associated with stage progression, delayed treatment, and worse outcomes, our finding that LDAP management significantly increases the likelihood of completing staging investigations and undergoing faster staging may in part explain our prior finding that LDAP management is associated with improved 2-year lung cancer survival. These findings underscore the critical role of coordinated and structured care pathways, such as the LDAP, in improving the efficiency and effectiveness of LC staging processes. The study also highlights the persistent challenges posed by geographic and logistical barriers, particularly the limited accessibility to PET-CT facilities, which need to be addressed to further enhance patient outcomes. Future initiatives should prioritize mitigating these barriers and expanding access to essential diagnostic resources to ensure timely and comprehensive care for all LC patients.

## Figures and Tables

**Table 1:** Definition of optimal and alternative staging.

Lung Cancer Stage	Optimal Staging	Alternative Staging
Stage I	PET-CT	CT abdomen + bone scan
Stage II	PET-CT +brain imaging * + invasive mediastinal staging **	CT abdomen + bone scan + brain imaging * + invasive mediastinal staging **
Stage III	PET-CT +brain imaging * + invasive mediastinal staging **	CT abdomen + bone scan + brain imaging * + invasive mediastinal staging **

* Brain imaging defined as brain MRI or head CT. ** Mediastinal staging defined as endobronchial ultrasound (EBUS) or mediastinoscopy.

**Table 2:** Patient and disease characteristics in accordance with completeness of staging investigations.

Characteristic	Staging Completeness				*p*-Value
	Total (N = 755)	Optimal (N = 596)	Alternative (N = 23)	Incomplete (N = 136)	
**Age**					
18–60 years	105 (13.9%)	76/105 (72.4%)	4/105 (3.8%)	25/105 (23.8%)	0.84
61–70 years	237 (31.4%)	198/237 (83.5%)	6/237 (2.5%)	33/237 (13.9%)	
71–80 years	292 (38.7%)	235/292 (80.5%)	9/292 (3.1%)	48/292 (16.4%)	
81+ years	121 (16%)	87/121 (71.9%)	4/121 (3.3%)	30/121 (24.8%)	
**Sex**					
Female	438 (58.0%)	354/438 (80.8%)	12/438 (2.7%)	72/438 (16.4%)	0.32
Male	317 (42.0%)	242/438 (76.3%)	11/317 (3.5%)	64/317 (20.2%)	
**Histology Type**					
Adenocarcinoma	329 (43.6%)	279/329 (84.8%)	7/329 (2.1%)	43/329 (13.1%)	**0.002**
Non-adenocarcinoma	425 (56.4%)	316/425 (74.4%)	16/425 (3.8%)	93/425 (21.9%)	
**Income Quintile**					
1 (lowest)	130 (17.2%)	100/130 (76.9%)	1/130 (0.8%)	29/130 (22.3%)	0.050
2	220 (29.1%)	165/220 (75.0%)	13/220 (5.9%)	42/220 (19.1%)	
3	250 (33.1%)	207/250 (82.8%)	5/250 (2.0%)	38/250 (15.2%)	
4	118 (15.6%)	91/118 (77.1%)	4/118 (3.4%)	23/118 (19.5%)	
5 (highest)	37 (4.9%)	33/37 (89.2%)	0/37 (0.0%)	4/37 (10.8%)	
**Stage**					
I	382 (50.6%)	327/382 (85.6%)	12/382 (3.1%)	43/382 (11.3%)	**<0.0001**
II/III	373 (49.4%)	269/373 (72.1%)	11/373 (2.9%)	9/373 (24.9%)	
**LDAP Managed**					
No	296 (39.2%)	205/296 (69.3%)	15/296 (5.1%)	76/296 (25.7%)	**<0.0001**
Yes	459 (60.8%)	391/459 (85.2%)	8/459 (1.7%)	60/459 (13.1%)	

Bold values indicate *p* < 0.05, highlighting statistically significant differences.

**Table 3:** Completeness of staging investigations according to staging test and distance.

Staging Test	Staging Completeness				
	Overall (N = 755)	Optimal (N = 596)	Alternative (N = 23)	Incomplete (N = 136)	*p*-Value
**Invasive mediastinal staging**	273 (36.2%)	229/273 (83.9%)	11/273 (4.0%)	33/273 (12.1%)	**0.004**
**EBUS**	231 (30.6%)	191/231 (82.7%)	10/231 (4.3%)	30/231 (13.0%)	**0.029**
**Mediastinoscopy**	52 (6.9%)	48/52 (92.3%)	1/52 (1.9%)	3/52 (5.8%)	**0.046**
**Brain imaging**	537 (71.1%)	450/537 (83.8%)	19/537 (3.5%)	68/537 (12.7%)	**<0.0001**
**Geodesic Distance to PET Centre (km)**					
<100	143 (18.9%)	139/143 (97.2%)	0/143 (0.0%)	4/143 (2.8%)	0.14
100–200	360 (47.7%)	357/360 (99.2%)	0/360 (0.01%)	3/360 (0.8%)	
>200	95 (12.6%)	91/95 (95.8%)	0/95 (0.0%)	4/95 (4.2%)	
Unknown/no PET	157 (20.8%)	9/157 (5.7%)	23/157 (14.6%)	125/157 (79.6%)	
**Travel distance to PET Centre (km)**					
<100	91 (12.1%)	88/91 (96.7%)	0/91 (0.0%)	3/91 (3.3%)	0.20
100–200	295 (39.1%)	291/295 (98.6%)	0/295 (0.0%)	4/295 (1.4%)	
>200	212 (28.1%)	208/212 (98.1%)	0/212 (0.0%)	4/212 (1.9%)	
Unknown/no PET	157 (20.8%)	9/157 (5.7%)	23/157 (14.6%)	125/157 (79.6%)	
**Travel distance to nearest PET Centre (km)**					
<100	116 (15.4%)	91/116 (78.4%)	2/116 (1.7%)	23/116 (19.8%)	0.80
100–200	547 (72.5%)	430/547 (78.6%)	19 /547 (3.5%)	98/547 (17.9%)	
>200	92 (12.2%)	75/92 (81.5%)	2/92 (2.2%)	15/92 (16.3%)	
**PET Centre**					
KMH Mississauga	45 (6.0%)	44/45 (97.8%)	0/45 (0.0%)	1/45 (2.2%)	0.46
MyHealth Mississauga	45 (6.0%)	44/45 (97.8)	0/45 (0.0%)	1/45 (3.1%)	
PMH Toronto	32 (4.2%)	31/32 (96.9%)	0/32 (0.0%)	1/32 (3.1%)	
St. Joseph’s Hamilton	1 (0.1%)	1/1 (100%)	0 (0.0%)	0/1 (0.0%)	
Sunnybrook Toronto	31 (4.1%)	30/31 (96.8%)	0/31 (0.0%)	1/31 (3.2%)	
TOH General	444 (58.8%)	437/444 (98.4%)	0/444 (0.0%)	7/444 (1.6%)	
Unknown/No PET	157 (20.8%)	9/157 (5.7%)	23/157 (14.6%)	125/157 (79.6%)	

Bold values indicate *p* < 0.05, highlighting statistically significant differences.

**Table 4:** Timeliness of diagnosis and staging investigations and completeness of staging.

Time Interval	Staging Completeness				
	Overall (N = 755)	Optimal (N = 596)	Alternative (N = 23)	Incomplete (N = 136)	*p*-Value
**Time to Diagnosis** (days)					
Mean (SD)	40.3 (29.2)	42.9 (27.1)	23.9 (31.9)	31.9 (35.2)	**<0.001**
Median (IQR)	37.0 (24.0, 55.0)	40.0 (28.0, 56.0)	26.0 (0.0, 51.0)	26.0 (4.0, 49.5)	
**Time to PET-CT**					
Mean (SD)	42.6 (29.1)	42.6 (29.2)	-	43.1 (21.2)	0.96
Median (IQR)	36.0 (26.0, 53.0)	36.0 (25.5, 53.0)	-	45.0 (26.0, 49.0)	
**Time to Staging Investigations**					
Mean (SD)	48.6 (31.7)	49.0 (31.5)	40.0 (36.8)	-	0.18
Median (IQR)	41.0 (28.0, 61.0)	41.0 (29.0, 62.0)	28.0 (16.0, 50.0)	-	

Bold values indicate *p* < 0.05, highlighting statistically significant differences.

**Table 5:** Factors associated with completeness of staging investigation for patients with stage I–III lung cancer—unadjusted and adjusted analyses.

Factor	Unadjusted		Adjusted	
	OR (95% CI)	*p*-Value	OR (95% CI)	*p*-Value
**Age**				
18–60	reference	**0.027**	reference	**0.048**
61–70	1.93 (1.08, 3.45)		1.66 (0.91, 3.01)	
71–80	1.59 (0.92, 2.74)		1.39 (0.79, 2.45)	
81+	0.95 (0.52, 1.74)		0.79 (0.42, 1.48)	
**Sex**				
Female	1.29 (0.89, 1.87)	0.19	reference	0.23
Male	reference		0.79 (0.54, 1.16)	
**Income Quintile**				
1 (lowest)	0.42 (0.14, 1.29)	0.34	0.48 (0.15, 1.51)	0.46
2	0.51 (0.17, 1.53)		0.54 (0.18, 1.68)	
3	0.68 (0.23, 2.02)		0.73 (0.24, 2.26)	
4	0.50 (0.16, 1.56)		0.56 (0.17, 1.80)	
5 (highest)	reference		reference	
**LDAP management**				
No	reference	**<0.0001**	reference	**<0.0001**
Yes	2.30 (1.58, 3.35)	2.29 (1.53, 3.41)		
**Distance to nearest PET centre (km)**				
<100	reference	0.80	reference	0.77
100–200	1.13 (0.68, 1.88)		0.82 (0.48, 1.41)	
>200	1.27 (0.62, 2.60)		0.84 (0.39, 1.81)	

Bold values indicate *p* < 0.05, highlighting statistically significant differences.

**Table 6:** Factors associated with time from CT scan to complete staging investigation (days) among stage I–III lung cancer patients—unadjusted and adjusted analyses.

Factor	Unadjusted		Adjusted	
	β (95% CI)	*p*-Value	β (95% CI)	*p*-Value
**Age**				
18–60	reference	0.48	reference	0.24
61–70	−6.90 (−15.59, 1.80)		−6.69 (−13.55, 0.18)	
71–80	−5.42 (−13.92, 3.09)		−4.49 (−11.32, 2.35)	
81+	−4.89 (−15.77, 5.99)		−1.80 (−10.37, 6.77)	
**Sex**				
Female	−3.19 (−8.85, 2.48)	0.27	3.07 (−2.25, 8.39)	0.063
Male	reference		reference	
**Income Quintile**				
1 (lowest)	7.60 (−7.50, 22.70)	0.72	8.73 (−3.16, 20.61)	0.64
2	3.24 (−11.01, 17.50)		8.10 (−3.19, 19.40)	
3	4.25 (−9.87, 18.37)		7.05 (−4.19, 18.28)	
4	1.03 (−14.03, 16.09)		5.90 (−6.14, 17.95)	
5 (highest)	reference		reference	
**LDAP managed**				
No	reference	**0.023**	reference	**<0.0001**
Yes	−6.63 (−12.36, −0.91)		−18.53 (−23.48, −13.57)	
**Stage**				
I	−4.95 (−10.50, 0.60)	0.080	−13.69 (−18.50, −8.88)	**<0.0001**
II/III	reference		reference	
**Histology**				
Adenocarcinoma	reference	**<0.0001**	reference	**0.013**
Non-adenocarcinoma	−11.16 (−16.65, −5.66)		−5.90 (−1.25, −10.55)	
**First diagnostic procedure**				
CT-guided biopsy	reference	**0.021**	reference	**0.026**
EBUS/ bronchoscopy	−7.73 (−13.74, −1.72)		−6.98 (−12.34, −1.62)	
Other	−8.52 (−18.20, 1.17)		−6.09 (−13.85, 1.67)	
**Time to diagnosis**	18.71 (16.18, 21.24)	**<0.0001**	21.63 (19.16, 24.11)	**<0.0001**
**Distance to PET**	2.38 (−3.64, 8.41)	0.44	8.95 (3.82, 14.07)	**0.0007**

Bold values indicate p < 0.05, highlighting statistically significant differences.

## Data Availability

The datasets generated during and/or analyzed during the current study are not publicly available, but are available from the corresponding author on reasonable request.
